# Expression of MAP2, GFAP, and calcium in CA3 area of a modified organotypic hippocampal slice culture treated with kainic acid

**DOI:** 10.12688/f1000research.126732.2

**Published:** 2023-05-25

**Authors:** Machlusil Husna, Kusworini Handono, Hidayat Sujuti, Aulanni'am Aulanni'am, Ettie Rukmigarsari

**Affiliations:** 1 Doctoral Program of Medical Science, Faculty of Medicine, Universitas Brawijaya, Malang, East Java, Indonesia; 2 Department of Neurology, Faculty of Medicine Universitas Brawijaya / dr. Saiful Anwar General Hospital, Malang, East Java, Indonesia; 3 Department of Clinical Pathology, Faculty of Medicine Universitas Brawijaya / dr. Saiful Anwar General Hospital, Malang, East Java, Indonesia; 4 Department of Ophthalmology, Faculty of Medicine Universitas Brawijaya / dr. Saiful Anwar General Hospital, Malang, East Java, Indonesia; 5 Department of Chemistry, Faculty of Sciences Universitas Brawijaya, Malang, East Java, Indonesia; 6 Faculty of Teaching and Education Sciences, Islamic University of Malang, Malang, East Java, Indonesia

**Keywords:** organotypic hippocampal slice culture, MAP2, GFAP, intracellular calcium, excitotoxicity, kainic acid

## Abstract

**Background:** Neurodegeneration due to neurotoxicity is one of the phenomena in temporal lobe epilepsy. Experimentally, hippocampal excitotoxicity process can occur due to kainic acid exposure, especially in the CA3 area. Neuronal death, astrocyte reactivity and increased calcium also occur in hippocampal excitotoxicity, but few studies have investigated immediate effect after kainic acid exposure. The organotypic hippocampal slice culture (OHSC) is a useful model for studying the neurodegeneration process, but there are still many protocol differences. In this study, minor modifications were made in the OHSC protocol.

**Methods:** OHSC was obtained from two healthy wild type Wistar rats aged P10. Healthy culture slices were obtained and lasted up to 10 days *in vitro* (DIV 10). Bath application of kainic acid for 48 hours in DIV 10 followed by observation of its initial effects on neurons, astrocytes, and calcium via the expression of MAP2, GFAP, and intracellular calcium, subsequently.

**Results:** After 48 h of kainic acid administration, there was a significant increase in intracellular calcium (p = 0.006 < α), accompanied by a significant decrease in MAP2 (p = 0.003 < α) and GFAP (p = 0.010 < α) expression.

**Conclusion:** These findings suggest early neuronal and astrocyte damage at the initial onset of hippocampal injury. This implies that astrocyte damage occurs early before an increase in GFAP that characterizes reactive astrogliosis found in other studies. Damage to neurons and astrocytes may be associated with increased intracellular calcium. It is necessary to develop further research regarding expression of calcium, MAP2, and GFAP at a spatial time after exposure to kainic acid and strategies to reduce damage caused by excitotoxicity.

## Introduction

Neurodegeneration mainly caused by acute and chronic excitotoxicity. The chronic excitotoxicity process can be found in Alzheimer’s disease (AD), Parkinson’s disease (PD), Huntington’s disease (HD), amyotrophic lateral sclerosis (ALS), and temporal lobe epilepsy (TLE) ( Doble, 1999; Mohd Sairazi *et al.*, 2015). The chemoconvulsant agent kainic acid (KA), which is an AMPA/kainate receptor agonist, is often used experimentally to induce excitotoxicity, as it plays a role in the development of epileptic seizures similar to conditions in epileptic patients ( Holopainen *et al.*, 2004; Tanioka *et al.*, 2020; Zhang & Zhu, 2011).

Neuronal death as a result of glutamate excitotoxicity has been extensively studied, both *in vivo* and *in vitro* ( Holopainen *et al.*, 2004; Liu *et al.*, 2001; Noraberg *et al.*, 1999; Sanon *et al.*, 2005; Sattler & Tymianski, 2001). Glutamate represents the primary excitatory neurotransmitter in the mammalian brain, and it is used by neurons, astrocytes, and other glial cells to process information ( Mihály, 2019). The main mechanism behind neuronal death is glutamate excitotoxicity ( Cho, 2013; Sanon *et al.*, 2005), which occurs through necrosis, apoptosis, or both ( Holopainen *et al.*, 2004; Liu *et al.*, 2001). Other than neuronal death, a significant increase in astrocyte reactivity has been observed in response to central nervous system injury ( Jeong *et al.*, 2014; Miller *et al.*, 2017; Yildirim *et al.*, 2019; Zhang *et al.*, 2017; Zhang & Zhu, 2011). Increased astrocyte reactivity (astrogliosis), characterized by increased GFAP expression, is known to exert a protective effect against neuronal death due to nervous system injury ( Jeong *et al.*, 2014; Miller *et al.*, 2017; Zhang *et al.*, 2017). Prior studies have found this process to be closely related to calcium level. In the context of epilepsy, the calcium hypothesis suggests an increase in intracellular calcium levels. If this increase is not sufficient to induce excitotoxicity, it can result in temporary or permanent neuroplasticity changes and degeneration. In the long term, these changes can cause abnormal neuronal firing and so lead to epilepsy ( Zhang *et al.*, 2019).

Kainic acid is commonly used for glutamate excitotoxicity model. Damage due to kainic acid mainly occurs in the hippocampus. Such damage may be caused by the high density of kainic receptors in that area ( Mihály, 2019; Zhang & Zhu, 2011). The CA3 circuit plays a role in the epileptiform activity within the hippocampus. Song *et al.* found hyperexcitability due to hypoxic-hypoglycemic injury to be more visible in the CA3 area than in other hippocampal areas ( Song *et al.*, 2018). The maturation of synapses, receptors, and intrinsic fiber pathways in the OHSC also occurs in *in-vivo* conditions, which renders it a useful model for studying age-related conditions in a maintained environment ( Holopainen *et al.*, 2004). To date, various methods have been used to make OHSCs. The preparation of acute hippocampal slices at room temperature gives rise to better results, especially with regard to electrophysiological observations ( Eguchi *et al.*, 2020). In contrast, OHSC processing requires cold temperatures during slicing in order to reduce the damage that may occur throughout the surgical process, although there are still many variations when it comes to the use of cold temperatures ( Lein *et al.*, 2011; Opitz-Araya & Barria, 2010; Simoni & Yu, 2006; Varela *et al.*, 2012).

Only a few prior studies have investigated the immediate impact of hippocampal insult to neuron and astrocyte ( Jeong *et al.*, 2014; Zhao *et al*., 2003). This study aimed to observe the immediate effect of kainic acid on neurons, astrocytes, and intracellular calcium in the CA3 area of modified OHSC.

## Methods

### Overview of experiment

This study used three healthy wild type Wistar rats aged postnatal day 10 (P10), weight range 10-15 grams, regardless of sex. The sample size was decided from Federer’s formula ( Ihwah *et al.*, 2018). The number of treatments are two, and the result from the formula is 8, so we used 9 slices for each subgroup. All of the Wistar rats were purchased from Animal Lab of Biosains Institute Universitas Brawijaya. All the procedures carried out in this study were approved by the Health Research Ethics Commission, Faculty of Medicine, Brawijaya University Malang (No.50/EC/KEPK-S3/02/2019).

We obtained approximately 18-20 hippocampal slices per rat and cultured them to make the appropriate organotypic hippocampal slice culture (OHSC; made at the Biosciences Institute of Universitas Brawijaya). The OHSC was divided into two groups (allocated randomly), namely the untreated group and the kainate group. The kainate group was treated with kainic acid (Abcam, AB120100) at a dose of 8 μM by bath application for 48 h and put in an incubator at 37 ^o^C. After that, both groups (kainate group and untreated group) were stained for calcium, MAP2, and GFAP (each subgroup consist of 9 slices) and observed with confocal laser scanning microscopy (CLSM). Sliced culture photographs were taken from a stereomicroscope observation at 40× magnification. The photos were processed with Adobe Photoshop ver. 2020. We examined the healthiness of the slices macroscopically, using stereomicroscope. The healthy slices will look thinner and more transparent, because the dead cells and tissue debris have disappeared. Another marker is we can see the different region of hippocampus clearly ( Simoni & Yu, 2006). On day *in vitro* 13 (DIV 13), some slices were not very healthy macroscopically, so we exclude some slices for statistical analysis. The excluded slices conditions are: not getting thinner and transparent, also the color become whitish opaque or more darker. Therefore, we used 5, 3 and 3 slices for calcium, MAP2, and GFAP, respectively. Parameter observations with CLSM were done in the Central Laboratory of Biological Sciences Universitas Brawijaya.

### Organotypic hippocampal slice culture procedure

The slicing medium and the culture medium were prepared before surgery. More specifically, the slicing medium was prepared using 71.49 g of HEPES (Sigma, H3375, Lot SLBB55267) in 300 ml of Earle’s Balanced Salt Solution (EBSS) 10X (Sigma, Lot RNBG8086). The culture medium comprised 50 ml of Minimum Eagle Medium (MEM, Sigma, M0769-10x1L, Lot SLBS6945), 18 ml of EBSS, 5 ml of EBSS + D-glucose (Merck, CAS-No: 50-99), 1 ml of penicillin-streptomycin (Gibco, Ref 15070-063, Lot 2145472), 25 ml of horse serum (Gibco, Ref 16050-122, Lot 2208948), and 0.06 ml of nystatin (Abcam, ab141118, Lot GR305852-5).

Age recommendation to make OHSC for rat is P0-15 and P0-5 for mice ( Muller *et al.*, 2001). We used P10 Wistar rat, because technically it was easier for us than younger rats. The brain size was not too small so it was easier to slice the hippocampus. The pups remained with the mother, mostly in a dark environment at a temperature of 26 ^o^C with 70% relative humidity. On P10 we took the pups for euthanasia. The OHSC was made according to the method proposed by de Simoni ( Simoni & Yu, 2006), albeit with slight modifications ( Opitz-Araya & Barria, 2010). Briefly, cervical dislocation followed by decapitation were performed aseptically and without any prior anesthesia ( Simoni & Yu, 2006). Cervical dislocation is appropriate and acceptable method of euthanasia for small rats weighing <200 grams. Cervical dislocation causes rapid loss of consciousness, and we performed decapitation immediately after cervical dislocation. Additionally the procedure was performed by a skilled technician and we require uncontaminated brain tissue since anesthetic drug can interfere brain properties ( Hollands, 1986; Karmarkar *et al.*, 2010; Leary *et al.*, 2020). The brain was removed from the skull and then soaked for 1 minute in a beaker placed on ice. The beaker contained a slicing medium that had been perfused with 5% CO _2_/95% O _2_ for 10 – 20 minutes. Next, the brain was transferred to a petri dish containing the slicing medium (2.4 g HEPES and 10 ml EBSS) and the hippocampus isolation was performed. Following the isolation, the next step involved making slices with a thickness of 350 μm using a tissue slicer (Stoelting Tissue Slicer 51425, RRID:SCR_022902). The hippocampal slices were then placed on an omnipore membrane (Merck, Ref: JHWP01300, Lot: R0MB18744) within a cell culture insert (Merck, Ref: PICM03050, Lot: R0DB59407) and a six-well plate filled with 1.3 ml of the culture medium (EBSS, glucose, penicillin, horse serum, Na bicarbonate, amphotericin, L-glutamine, EBSS, and MEM). All slices that were ready for culturing were put into an incubator at 37 ^o^C for 10 days. The culture medium was changed every two days.

The full protocol for this procedure can be found here: http://dx.doi.org/10.17504/protocols.io.81wgby6pnvpk/v1.

### Immunohistochemical staining procedure

The staining of microtubule-associated protein-2 (MAP2 ) (GeneTex Cat# GTX50810, RRID: AB 11170769) and glial fibrillary acidic protein (GFAP) (Santa Cruz Biotechnology Cat# sc-33673, RRID:AB_627673) were done at DIV 10 via the following procedure. Tissue slices were fixed using 4% paraformaldehyde (PFA) for 15 min, then washed with phosphate buffered saline (PBS) for three times (5 minutes each). After the PBS was removed, 0.1% Triton X-100 was added for 30 min, and the slice culture was washed with PBS for three times (5 minutes each). After washing, 10% bovine serum albumin (BSA) was added for 30 min at room temperature. The primary antibody incubation MAP2 (1: 500) and GFAP (1: 500) was performed overnight at 4°C, then washed with PBS for three times (5 minutes each). An incubation using a secondary antibody, Goat anti-Rabbit IgG-F (1: 500) for MAP2 (Santa Cruz Biotechnology Cat# sc-53805, RRID:AB_783978) and anti-mouse Rhodamine (1: 500) for GFAP (Rockland Cat# 610-1002, RRID:AB_219636) for 1 h at room temperature. It was subsequently washed with PBS for three times (5 minutes each), and the sample was ready to be observed using CLSM with a magnification of 100× and a wavelength of 488 nm (MAP2) and 543 nm (GFAP).

The full protocol for this procedure can be found here: http://dx.doi.org/10.17504/protocols.io.q26g7y4b1gwz/v1

### Intracellular calcium tracking

Fluo-4 was used for intracellular calcium tracking (Abcam Fluo-4 assay unit (calcium), AB228555). Nine mL of HHBS + 1 mL 10X F127 were mixed into a 15 mL conical tube, 20 μL of Fluo-4 was added. It was mixed in a dark room until homogeneous. Next, 100 μL of Fluo-4 AM dye-loading solution was added in a petri cell dish, followed by incubation in a cell incubator for 1 h. The observation of calcium fluorescence used CLSM at 100× magnification with the fluorescence intensity monitor at maximum Ex/Em = 490/525 nm.

The full protocol for this procedure can be found here: http://dx.doi.org/10.17504/protocols.io.kxygx9zewg8j/v1.

### Statistical analysis

The data normality test was performed using the Shapiro-Wilk test. Normally distributed data in both groups were compared using the independent sample t-test. Significant differences in the data were indicated by a p < 0.05. Statistical analysis was performed with SPSS for Windows 25.0 (SCR_016479).

## Results

**Figure 1.**
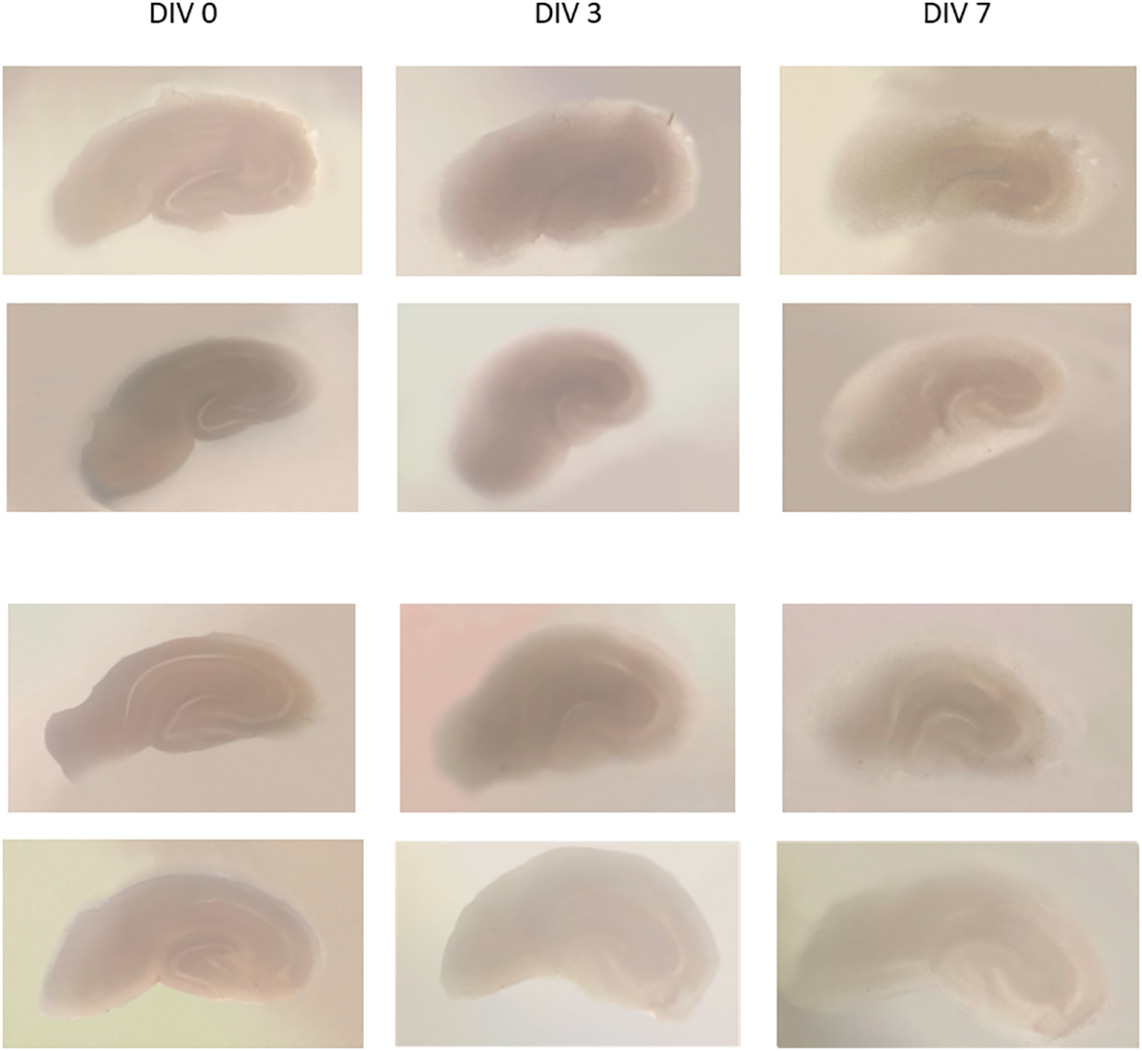
Some examples of hippocampal slice cultures of DIV 0, DIV 3, and DIV 7. It appears that the slices are thinner and become transparent, with parts of the hippocampus still visible. DIV = day *in vitro.*

Examples of the organotypic hippocampal slice culture results using a modified method from de Simoni ( Simoni & Yu, 2006) and Opitz-Araya ( Opitz-Araya & Barria, 2010) can be seen in Figure 1. Images of several slices of culture were taken at DIV 0, 3, and 7. On DIV 0, slices were seen dark-colored, and became more transparent and thinner on the 3rd and 7th day; the hippocampal architecture still visible. The slice thickness at DIV 0 was 350 μm, and the thickness at DIV 13 was about 150-200 μm, estimated by CLSM.

**Figure 2.**
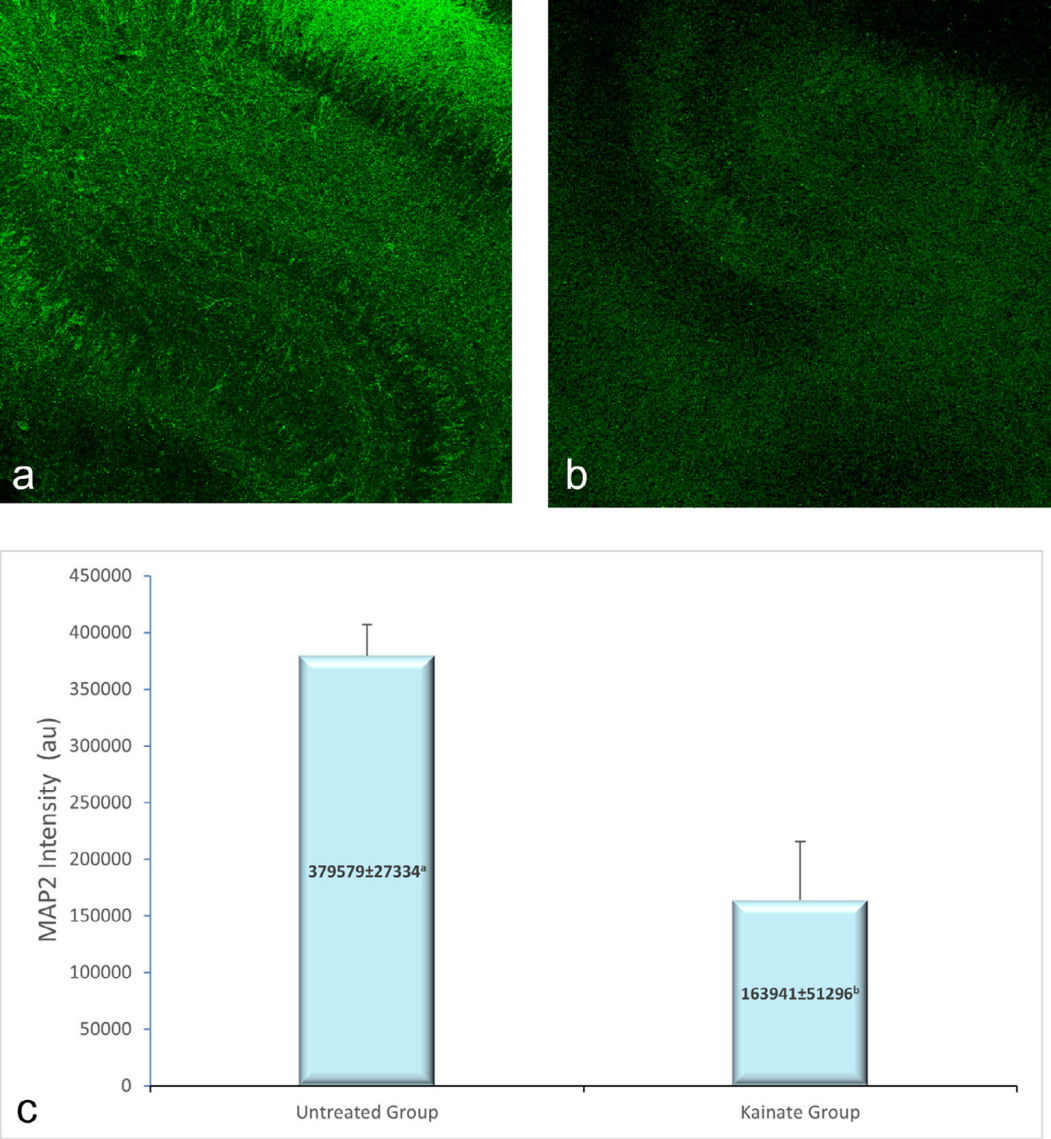
CLSM image of MAP2 expression in CA3 area. Comparison of MAP2 expression in the untreated group (a) and kainate group (b). c. There was a significant decrease of MAP2 expression in kainate group (p = 0.003 < α ). MAP2 = microtubule-associated protein 2. The denomination of expression intensity uses the arbitrary unit (AU).

**Figure 3.**
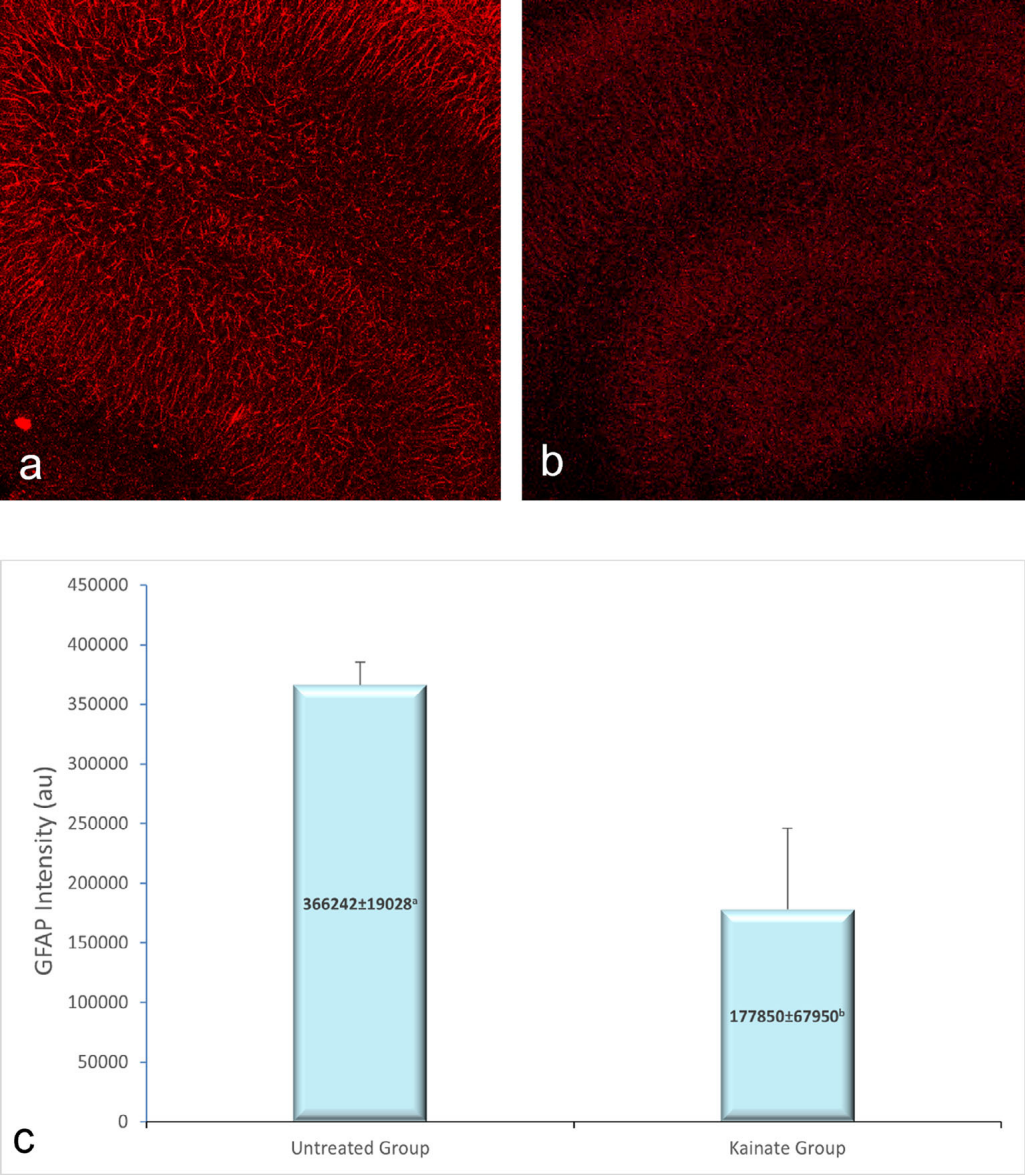
CLSM image of GFAP expression in CA3 area. Comparison of GFAP expression in the untreated group (a) and kainate group (b). c. There was a significant decrease of GFAP expression in kainate group (p = 0.010 < α ). GFAP = glial fibrillary acidic protein. The denomination of expression intensity uses the arbitrary unit (AU).

**Figure 4.**
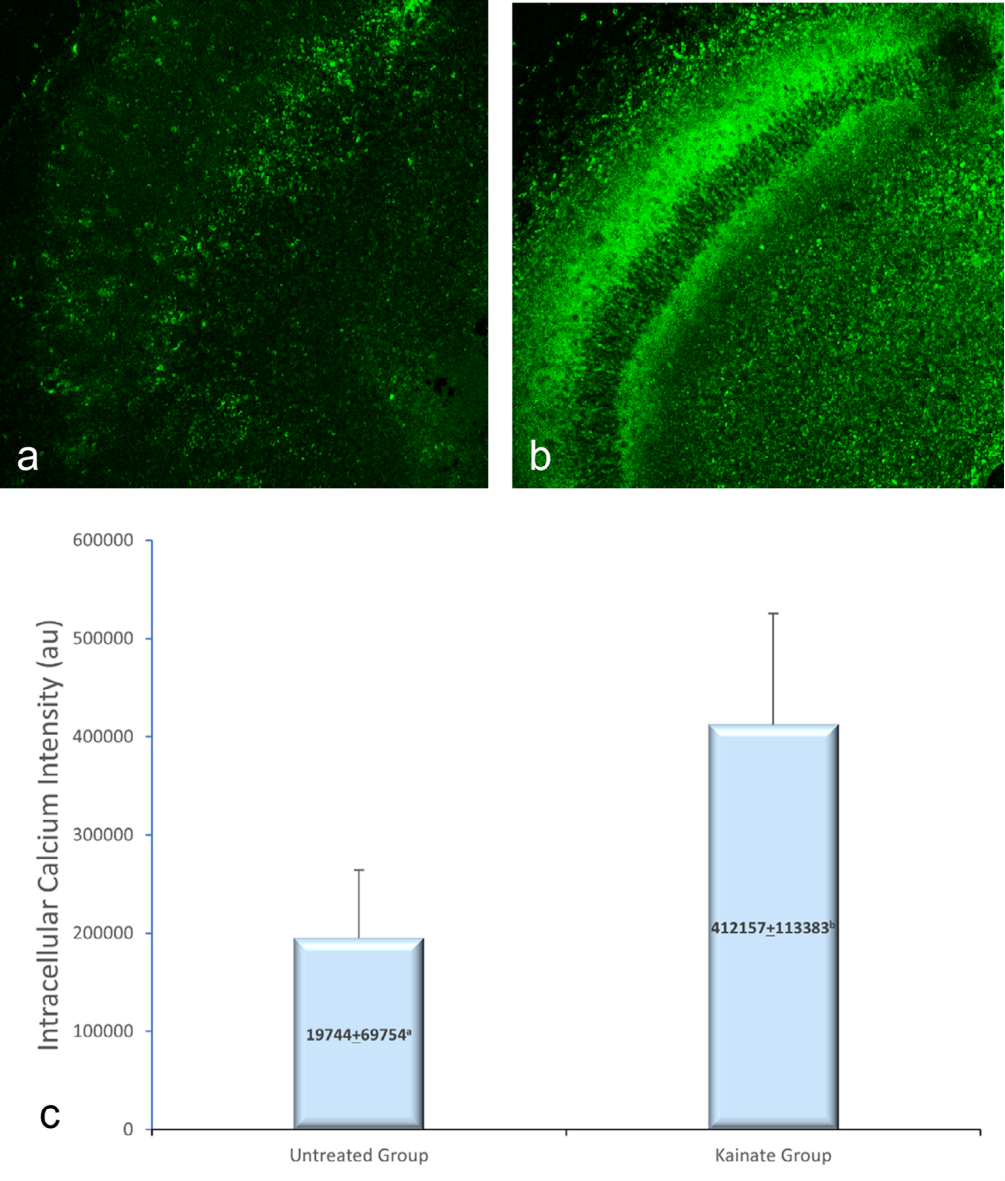
CLSM image of calcium expression in CA3 area. Comparison of calcium expression intensity in the untreated group (a) and kainate group (b). c. There was a significant increment of intracellular calcium expression in kainate group (p = 0.006 < α). The denomination of expression intensity uses the arbitrary unit (AU).

CLSM image of MAP2 expression in untreated and treated group shown in Figure 2(a-b), GFAP expression in Figure 3(a-b), and intracellular calcium expression in Figure 4(a-b). The mean intensity of MAP2, GFAP, and intracellular calcium expression in the untreated and kainate groups can be seen in Figures 2- 4(c). MAP2 ( Figure 2c) and GFAP ( Figure 3c) expression decreased significantly in the kainate group compared to the untreated group. There was a significant increase of intracellular calcium expression in treated versus untreated group ( Figure 4c).

Based on the results of the Shapiro-Wilk test, it was found that the data on calcium, MAP2, and GFAP intensities in the two observation groups had a p-value more significant than the significance level α = 0.05, which means that all the observation groups proved to be normally distributed.

The comparison test between the untreated and the kainate group using the independent sample t-test is shown in the Table 1.

**Table 1.** Results of comparisons between groups.

Variable	Untreated group Mean ± standee	Kainate group Mean ± standee	*p-value*
Intracellular calcium expression (AU)	194744 ± 69754	412157 ± 113383	0.006
MAP2 expression (AU)	379579 ± 27334	163941 ± 51296	0.003
GFAP expression (AU)	366242 ± 19028	177850 ± 67950	0.010

**Notes:** p-value < 0.05 means that there is a significant difference, and p-value > 0.05 means there is no significant difference.

Mean of MAP2 intensity in the kainate group was significantly lower than the untreated group (379579 ± 27334 AU vs 163941 ± 51296 AU; p = 0.003 < α). Likewise, we found significant difference in GFAP expression (p = 0.010 < α) between untreated group (366242 ± 19028 AU) and kainate group (177850 ± 67950 AU).

There is a significant difference (p = 0.006 < α) of intracellular calcium expression in kainate group (412.157 ± 113.383 AU) compared to untreated group (194.744 ± 69.754 AU). This means that the treated culture exposed to 8 μM of kainic acid for 48 h shows an increased intracellular calcium.

## Discussion

The OHSC protocol according to de Simoni, has been widely used ( Abutbul *et al.*, 2012; Kang *et al.*, 2010; Stephen *et al.*, 2015), as well as the method of Opitz-Araya ( McQuate & Barria, 2020; Park *et al.*, 2020; Yang *et al.*, 2020). The two methods are mainly similar, however, Opitz-Araya immersed the brain in the slicing medium with perfusion before performing hippocampus isolation ( Opitz-Araya & Barria, 2010). Low temperature is required to decrease metabolic activity ( Eguchi *et al.*, 2020), glutamate release, excitotoxicity, and purify the blood to aid in the visualization of the cell in slices. Perfusion of the slicing medium with a mixture of 95% O _2_ gas and 5% CO _2_ aimed to reduce the hypoxic-ischemic damage to the brain that can occur between decapitation time and the brain's placement in the medium ( Varela *et al.*, 2012). Combining the two methods produced the desired results, and the culture was viable until the 10th day. The culture slices were thinner and more transparent compared to day 0. This is an indicator of culture health macroscopically ( Opitz-Araya & Barria, 2010; Simoni & Yu, 2006). We could not use the unhealthy slices, so we exclude them for statistical analysis. We used only the healthy ones. The minimum requirement for comparation analysis is triplicate (we used 5 for calcium, 3 for MAP2, and 3 for GFAP analysis). The statistical analysis still can be done, and the result was significant, in accordance with the visual image obtained.

The three main findings of this study were significant decrease of MAP2 and GFAP expression, which are indicators of neuronal and astrocyte injury in the CA3 area, accompanied by a significant increase in intracellular calcium expression at the same time, that is, after 48 h of exposure to kainic acid. MAP2 is a cytoskeletal protein located in the perikaryal and dendrites that specific for neurons, functions for microtubule stabilization, and participates in the dendrite branching processes during the development of hippocampal neurons. This molecule is thought to play a role in synaptic plasticity mechanisms ( Kim *et al.*, 2020; Yildirim *et al.*, 2019), which also occurs in epilepsy ( Pitkänen & Lukasiuk, 2011). Expression of the MAP2 protein is primarily regulated during brain development and maturation ( Blümcke *et al.*, 2001). These proteins are frequently used as markers of dendrites and neuron maturity and integrity ( Melani *et al.*, 2005; Pitkänen & Lukasiuk, 2011). The decrease in expression of MAP2 in this study is appropriate with prior studies on OHSC ( Holopainen *et al.*, 2004; Hoskison *et al.*, 2007; Noraberg *et al.*, 1999). One study reported neuronal death in CA3 areas, especially after 12, 24, and 48 hours of 5mM KA exposure. The highest neuron mortality was found on exposure to KA for 48 hours, with necrosis as the underlying death process ( Holopainen *et al.*, 2004). Other researchers showed that neurons could still be observed within 3 hours after KA injection into the mice's cortex even though they did not appear healthy morphologically. On day 7, neurons disappeared in the entire hemisphere, indicating delayed neuronal death, supporting the apoptotic process ( Jeong *et al.*, 2014). Hoskinson *et al.* demonstrated that loss of MAP2 dendrite in CA1 areas is an indicator of excitotoxic damage after neuronal injury, both *in vivo* and *in vitro*, this phenomenon is dependent on Ca ^2+^ permanently ( Hoskison *et al.*, 2007). Lopim *et al.* found that the number of hippocampal neurons in epileptic mice decreased compared to normal mice and that the total number of neurons was negatively correlated with the frequency of epileptic seizures. The number of cell deaths occurred in the initial period after the seizure onset ( Lopim *et al.*, 2016).

Astrocytes secrete several inhibitory factors in conditions of injury, ischemia, blood-brain barrier damage, inflammatory reactions, abnormal metabolism, and oxidative stress ( Zhang *et al.*, 2017). The active inflammatory status in astrocytes is characterized by the upregulation of GFAP ( Siracusa *et al.*, 2019). These external factors influence axon regeneration and recovery of CNS neurological function. GFAP is one of the best markers for astrocyte activation due to injury or stress to the CNS ( Zhang *et al.*, 2017). During development, astrocytes also play a role in synaptic transmission, differentiation and migration of neurons, and axon growth. GFAP is an intermediate protein filament expressed in mature astrocytes. This protein is vital for adaptation to neuronal changes and activity during brain development. GFAP also plays a role in neural-glial interactions. Changes in GFAP levels can cause damage to the relationship between neurons and between neurons and glial cells ( Yildirim *et al.*, 2019). Jeong *et al.* found that astrocytes swelled within 3 hours of KA injection into the mice cortex. After that, GFAP+ astrocytes began to disappear from day one and increased until day 7. The location of the loss of astrocytes is the same as delayed neuronal death, thus supporting the suggestion that astrocytes may play a role in the onset of delayed neuronal death ( Jeong *et al.*, 2014; De Pablo *et al.*, 2013). The decreased expression of MAP2 after exposure to kainic acid in this study indicated damage to neurons due to administration of kainic acid.

This study found that kainic acid caused early damage to neurons and astrocytes, particularly at the onset of exposure. Astrocyte activation, characterized by increased GFAP expression, inhibits the inflammatory response after injury, limiting cell damage ( Zhang *et al.*, 2017). Decreased astrocyte activation in GFAP ^-/-^Vim ^-/-^ mice with an ischemic stroke resulted in larger infarct size. This implies that astrocyte activation is essential for protecting brain tissue in stroke and that GFAP plays a role in this process, especially in the cell resistance to oxidative stress ( De Pablo *et al.*, 2013). In this study, the decreased expression of GFAP after exposure to kainic acid indicated damage to astrocytes due to administration of kainic acid.

As previously mentioned, kainic acid causes excessive activation of glutamate receptors, leading to excitotoxicity ( Cho, 2013; Mohd Sairazi *et al.*, 2015; Sanon *et al.*, 2005). Vargas *et al.* reported that ionotropic glutamate receptors expression was common in young mice's slice cultures (mean age P10). In the adult hippocampus, astrocytes expressed KAR (kainate receptor) after the status epilepticus induced by kainic acid, especially GluK1. This increase is thought to be related to astrocytes' function as glutamate sensors after status epilepticus, as an attempt to save neurons from injury. Besides, KAR expression is also thought to increase calcium signal and hyperexcitability, which causes epileptic seizures ( Vargas *et al.*, 2013). A study reported a loss of GFAP immunoreactivity within 24 hours of intracortical injection of kainic acid in mice. This loss of GFAP precedes neuronal death ( Jeong *et al.*, 2014). Loss of GFAP immunoreactivity at the onset of traumatic brain injury has also been reported ( Zhao *et al.*, 2003).

The role of astrocytes in changes in neuronal activity is found in epilepsy. Astrocytes can trigger the induction and development of inflammatory status via Ca ^2+^ signaling and are closely related to disease grading. Ca ^2+^ variations that affect neuronal activity in releasing gliotransmitters are also one of the astrocytes' roles. The glutamate transporter is found in several neuron cell types, and astrocytes primarily play a role in glutamate uptake. GLT-1 is a glutamate transporter found in astrocytes, which plays a role in removing extracellular glutamate and increasing epileptogenic foci levels. Glutamine synthetase was reduced in the hippocampus of TLE patients compared to healthy patients. This downregulation causes glutamate-glutamine circulation and increases the accumulation of transmitters in the extracellular space and astrocytes. This indicates a condition of hyperexcitability that depends on astrocytes. AMPA receptors, especially the subtypes formed by the GLuR1 to GluR4 sub-units, are highly expressed in astrocytes. Epilepsy patients show increased expression of the GluR1 variant, which causes prolonged receptor opening, thereby increasing the influx of Na ^+^ and Ca ^2+^ ions, blocking astroglia Kir channels, increasing depolarization, and reducing the capacity of astrocytes in the K ^+^ ion buffer ( Siracusa *et al.*, 2019).

The third finding in this study was an increase in intracellular calcium expression after exposure to kainic acid. OHSC is an *in vitro* preparation used to study intact neural circuits in chronic conditions, including epileptogenesis. After tissue incision makes deafferentation, the neural tissue rearranges itself and exhibits epileptiform-like activity, which can be visualized by a calcium indicator. This calcium activity is present in both astrocytes and neurons ( Gee *et al.*, 2015). Damage to the CA3 area is due to direct stimulation of the kainate receptors, increased glutamate efflux ( Zhang & Zhu, 2011), and increased calcium ( Borges *et al.*, 2006). KA binding to its receptors increases cell depolarization, one of the main phenomena in temporal lobe epilepsy. Epileptogenesis is associated with increased glutamate neurotransmission. Glutamate triggers an increase in cytoplasmic free Ca ^2+^, which will increase the release of glutamate from neurons and astrocytes ( Zhang *et al.*, 2019). Intracellular calcium plays an essential role in regulating cell function ( Kurniawan, 2015). The intracellular accumulation of Ca ^2+^ also triggers a molecular cascade involving several intracellular messenger systems, which results in neuronal death. The amplitude of calcium transient in CA1, CA3, and dentate gyrus (DG) after KA administration was significantly higher than before KA administration. Kainic acid receptors were widely expressed in the hippocampus, presynaptically and postsynaptically. The KA1 sub-unit mostly found in the CA3 area and a little in the CA1 area, while the KA2 sub-unit is expressed equally in both the CA1 and CA3 areas ( Zhang *et al.*, 2019). Excitotoxicity is the leading cause of cell death, in this case, due to neurotransmission disturbances caused by excessive glutamate. Consequently, this triggers an increase in calcium and causes cell death and neuronal degeneration ( Ghibelli *et al.*, 2010). The increase in intracellular calcium influx and oxidative stress due to excessive activation of glutamate receptors by KA causes cell damage and increases in protease and endonuclease enzymes that damage membranes, cytoskeletal proteins, and DNA fragmentation. This oxidative stress plays a role in the death of neurons and glial cells ( Mohd Sairazi *et al.*, 2015). The significant increase in intracellular calcium expression in the kainate group implies that calcium plays a significant role in neuron and astrocyte damage due to kainic acid administration.

The strength of this study is the demonstration of early damage of neuron and astrocyte by kainic acid, accompanied with high intracellular calcium expression. Only few prior studies have investigated it together. This early damage could lead to epileptogenesis. However, we only measure the effect of excitotoxicity at one time only. Therefore, this study’s results can still be developed to further determine the course of neurons, astrocytes, as well as calcium in the epileptogenesis process and to find strategies to minimize excitotoxicitiy.

## Conclusions

The reduction of MAP2 and GFAP expression in this study indicates the loss of neurons and also astrocytes at the initial onset of exposure to kainic acid, which can be analogous to the deterioration of neuron and astrocyte function at the onset of insult leading to epilepsy.

## Data Availability

Figshare: Expression of MAP2, GFAP, and Calcium in CA3 Area of a Modified Organotypic Hippocampal Slice Culture Treated with Kainic Acid, https://doi.org/10.6084/m9.figshare.21367596.v3 ( Husna *et al.*, 2022). This project contains the following underlying data:
1. UNT CA SLICE B (.png) = Calcium Intensity Untreated Group 2. UNT CA SLICE C (.png) = Calcium Intensity Untreated Group 3. UNT GFAP SLICE B (.png) = GFAP Expression Untreated Group 4. UNT MAP 2 SLCIE B (.png) = MAP 2 Expression Untreated Group 5. AK8 CA SLICE B (.png) = Calcium Intensity Kainate Group 6. AK8 CA SLICE C (.png) = Calcium Intensity Kainate Group 7. AK8 GFAP SLICE B (.png) = GFAP Expression Kainate Group 8. AK8 GFAP SLICE C (.png) = GFAP Expression Kainate Group 9. AK8 MAP 2 SLICE B (.png) = MAP 2 Expression Kainate Group 10. AK8 MAP 2 SLICE C (.png) = MAP 2 Expression Kainate Group 11. MAP 2Untreated (.tif ) = MAP 2 Expression Untreated Group 12. MAP 2Kainate (.tif ) = MAP 2 Expression Kainate Group 13. GFAPKainate (.tif ) = GFAP Expression Kainate Group 14. GFAPUntreated (.tif ) = GFAP Expression Untreated Group 15. CalciumKainate (.tif ) = Calcium Intensity Kainate Group 16. CalciumUntreated (.tif ) = Calcium Intensity Untreated Group 17. MAP 2Kainate300 (.tif ) = MAP 2 Expression Kainate Group (300 dpi) 18. MAP 2Untreated300 (.tif ) = MAP 2 Expression Untreated Group (300 dpi) 19. GFAPKainate300 (.tif ) = GFAP Expression Kainate Group (300 dpi) 20. GFAPUntreated300 (.tif ) = GFAP Expression Untreated Group (300 dpi) 21. CalciumKainate300 (.tif ) = Calcium Intensity Kainate Group (300 dpi) 22. CalciumUntreated300 (.tif ) = Calcium Intensity Untreated Group (300 dpi) 23. DIV0-1 (.tif ) = Hippocampal Slice Culture Day in vitro 0 24. DIV0-2 (.tif ) = Hippocampal Slice Culture Day in vitro 0 25. DIV0-3 (.tif ) = Hippocampal Slice Culture Day in vitro 0 26. DIV0-4 (.tif ) = Hippocampal Slice Culture Day in vitro 0 27. DIV0-1300 (.tif ) = Hippocampal Slice Culture Day in vitro 0 (300 dpi) 28. DIV0-2300 (.tif ) = Hippocampal Slice Culture Day in vitro 0 (300 dpi) 29. DIV0-3300 (.tif ) = Hippocampal Slice Culture Day in vitro 0 (300 dpi) 30. DIV0-4300 (.tif ) = Hippocampal Slice Culture Day in vitro 0 (300 dpi) 31. DIV3-1 (.tif ) = Hippocampal Slice Culture Day in vitro 3 32. DIV3-2 (.tif ) = Hippocampal Slice Culture Day in vitro 3 33. DIV3-3 (.tif ) = Hippocampal Slice Culture Day in vitro 3 34. DIV3-4 (.tif ) = Hippocampal Slice Culture Day in vitro 3 35. DIV3-1300 (.tif ) = Hippocampal Slice Culture Day in vitro 3 (300 dpi) 36. DIV3-2300 (.tif ) = Hippocampal Slice Culture Day in vitro 3 (300 dpi) 37. DIV3-3300 (.tif ) = Hippocampal Slice Culture Day in vitro 3 (300 dpi) 38. DIV3-4300 (.tif ) = Hippocampal Slice Culture Day in vitro 3 (300 dpi) 39. DIV7-1 (.tif ) = Hippocampal Slice Culture Day in vitro 7 40. DIV7-2 (.tif ) = Hippocampal Slice Culture Day in vitro 7 41. DIV7-3 (.tif ) = Hippocampal Slice Culture Day in vitro 7 42. DIV7-4 (.tif ) = Hippocampal Slice Culture Day in vitro 7 43. DIV7-1300 (.tif ) = Hippocampal Slice Culture Day in vitro 7 (300 dpi) 44. DIV7-2300 (.tif ) = Hippocampal Slice Culture Day in vitro 7 (300 dpi) 45. DIV7-3300 (.tif ) = Hippocampal Slice Culture Day in vitro 7 (300 dpi) 46. DIV7-4300 (.tif ) = Hippocampal Slice Culture Day in vitro 7 (300 dpi) 47. Calcium (.tif ) = Calcium Intensity Graphic 48. GFAP (.tif ) = GFAP Expression Graphic 49. MAP 2 (.tif ) = MAP 2 Expression Graphic 50. Calcium600 (.tif ) = Calcium Intensity Graphic (600 dpi) 51. GFAP600 (.tif ) = GFAP Expression Graphic (600 dpi) 52. MAP 2600 (.tif ) = MAP 2 Expression Graphic (600 dpi) Data are available under the terms of the Creative Commons Attribution 4.0 International license (CC-BY 4.0).
