# Supplementary material for: Regulation of MAP2, GFAP, and calcium in the CA3 Region Following Kainic Acid Exposure to  organotypic hippocampal slice culture
Source: F1000Res. 2025 Jan 3;12:47. Originally published 2023 Jan 12. [Version 3] doi: 10.12688/f1000research.126732.3 (PMC11733419; doi:10.12688/f1000research.126732.3)
Supplement: Peer review history

# Reviewer report: Reviewer response for version 3

**DOI:** 10.5256/f1000research.176262.r355695

**Published:** 2025-01-14

**Authors:** Amin Derouiche

**Affiliations:** 1 University of Frankfurt, Frankfurt, Germany

I have now looked at Version 3 of this MS and advance its status to fully approved.

Is the work clearly and accurately presented and does it cite the current literature?

Yes

If applicable, is the statistical analysis and its interpretation appropriate?

Yes

Are all the source data underlying the results available to ensure full reproducibility?

No source data required

Is the study design appropriate and is the work technically sound?

Partly

Are the conclusions drawn adequately supported by the results?

Partly

Are sufficient details of methods and analysis provided to allow replication by others?

Yes

Reviewer Expertise:

morphology, immunocytochemistry, astrocyte, hippocampus

I confirm that I have read this submission and believe that I have an appropriate level of expertise to confirm that it is of an acceptable scientific standard.

# Reviewer report: Reviewer response for version 2

**DOI:** 10.5256/f1000research.148364.r334920

**Published:** 2024-11-08

**Authors:** Amin Derouiche

**Affiliations:** 1 University of Frankfurt, Frankfurt, Germany

Expression of MAP2, GFAP, and calcium in CA3 area of a modified organotypic hippocampal slice culture treated with kainic acid

 Husna M, Handono K, Sujuti H et al.

 The study is well written. Since the issue has been well studied and documented – as cited by the authors – I wonder however what the study really reports. Is it the slice culture modification, is it the focus on the CA3 region, the immediacy of effect, or the antigen expression? This should be clarified in Title and Abstract, please change accordingly. The authors should be more clear in particular in relation to Jeong et al., 2014; De Pablo et al., 2013; and Holopainen et al., 2004.

 The Discussion is lengthy, the long paragraph at its end should be omitted altogether (“The third finding in this study […] death of neurons and glial cells ().”) The subsequent conclusions related to calcium are fully sufficient (“The significant increase […] leading to epilepsy.”)

 MINOR

- p. 9, end of first para.: “The number of cell deaths occurred in the initial period …” – please more preceise, when?
- Top para. p. 10: “receptor opening” – please replace.
- Last Discussion paragraph: “Only few prior studies have investigated it altogether.” Please supply references.
- How many animals were sacrificed for this study, three (Methods, first line) or two (Abstract)?
- Methods: “… we used 9 slices for each subgroup.” and “..we used 5,3, and 3 slices for calcium, MAP2, and GFAP, respectively” – can this be reconciled? please clarify.
- Analysis of Calcium conc. is interesting, but Fluo4 should not be described as “calcium staining”, or “calcium expression”; also “intracellular calcium tracking” is inappropriate since resolution is very low. “Low resolution calcium imaging” would be fine throughout.
- In all bar diagrams, please indicate “n=x”under each bar.
- Since no in vivo experimants were carried out, please consider replacing “epileptogenesis”and “epilepsy” by “epileptiform activity” and “seizure”, respectively.

Is the work clearly and accurately presented and does it cite the current literature?

Yes

If applicable, is the statistical analysis and its interpretation appropriate?

Yes

Are all the source data underlying the results available to ensure full reproducibility?

No source data required

Is the study design appropriate and is the work technically sound?

Partly

Are the conclusions drawn adequately supported by the results?

Partly

Are sufficient details of methods and analysis provided to allow replication by others?

Yes

Reviewer Expertise:

morphology, immunocytochemistry, astrocyte, hippocampus

I confirm that I have read this submission and believe that I have an appropriate level of expertise to confirm that it is of an acceptable scientific standard, however I have significant reservations, as outlined above.

# Response

**Published:** 2024-12-20

**Authors:** Machlusil Husna

**Affiliations:** Neurology Department, Faculty of Medicine, Universitas Brawijaya Malang, Indonesia

We thank the reviewer for his insightful feedback and constructive suggestions. Below, we provide a point-by-point response and the changes made to the manuscript

  

1. The study is well written. Since the issue has been well studied and documented – as cited by the authors – I wonder however what the study really reports. Is it the slice culture modification, is it the focus on the CA3 region, the immediacy of effect, or the antigen expression? This should be clarified in Title and Abstract, please change accordingly.
  - We agree with the reviewer that the focus of the study requires clarification. The title and abstract of the manuscript have been revised to emphasize the novel aspect of the study : Regulation of MAP2, GFAP, and Intracellular Calcium in the CA3 region following kainic acid exposure to organotypic hippocampal slice culture.
2. The authors should be more clear in particular in relation to Jeong et al., 2014; De Pablo et al., 2013; and Holopainen et al., 2004.
  - We have revised the introduction and discussion section to explicitly differentiate the findings from those of Jeong et al., 2014; De Pablo et al., 2013; and Holopainen et al., 2004
3. The Discussion is lengthy, the long paragraph at its end should be omitted altogether (“The third finding in this study […] death of neurons and glial cells ().”) The subsequent conclusions related to calcium are fully sufficient (“The significant increase […] leading to epilepsy.”)
  - The final paragraph of the discussion altogether (“The third finding in this study […] death of neurons and glial cells ().”) has been removed as suggested.  We keep the first sentence as the conclusions about calcium-related findings have been retained and strengthened
4. p. 9, end of first para.: “The number of cell deaths occurred in the initial period …” – please more precise, when?
  - We have added the initial period at 30 d after the seizure onset, in accordance with the reference
5. Last Discussion paragraph: “Only few prior studies have investigated it altogether.” Please supply references
  - We have added reference to the sentence as suggested
6. How many animals were sacrificed for this study, three (Methods, first line) or two (Abstract)?
  - The correct number is three. Thank you for the correction. We have revised the number of animal in abstract
7. Methods: “… we used 9 slices for each subgroup.” and “..we used 5,3, and 3 slices for calcium, MAP2, and GFAP, respectively” – can this be reconciled? please clarify.
  - The number of samples employed in each subgroup, specifically those pertaining to calcium, MAP2, and GFAP, was nine slices per group. These slices were cultured for 13 days, and upon staining at DIV 13, some slices were deemed unsuitable due to health concerns and thus excluded. Consequently, the final number of samples utilized for calcium imaging was five slices, for MAP2 was three slices,   and three slices for GFAP.
  - The writing has been refined in order to provide a more accurate and coherent representation of the intended meaning
8. Analysis of Calcium conc. is interesting, but Fluo4 should not be described as “calcium staining”, or “calcium expression”; also “intracellular calcium tracking” is inappropriate since resolution is very low. “Low resolution calcium imaging” would be fine throughout
  - We are grateful for your suggestion and  have  revised the terminology throughout the manuscript to low resolution calcium imaging”, in accordance with your advice.
9. In all bar diagrams, please indicate “n=x”under each bar.
  - We are grateful for your suggestion and have updated all bar diagrams to include sample sizues (n=x) under each bar,  in accordance with your advice
10. Since no in vivo experiments were carried out, please consider replacing “epileptogenesis”and “epilepsy” by “epileptiform activity” and “seizure”, respectively
  - We are  grateful for your suggestion and these terms have been replaced throughout the manuscript to reflect the in vitro nature of the study, in accordance with your advice

We hope these revision address the reviewer’s concerns and improve the manuscript’s clarity and focus. We are grateful for the reviewer's valuable feedback

# Reviewer report: Reviewer response for version 2

**DOI:** 10.5256/f1000research.148364.r175249

**Published:** 2023-06-13

**Authors:** Abdul Gofir

**Affiliations:** 1 Department of Neurology, Faculty of Medicine, Public Health and Nursing, Dr. Sardjito General Hospital, Universitas Gadjah Mada, Yogyakarta, Special Region of Yogyakarta, Indonesia

Overall this manuscript fulfil the revision suggested by the latest review. The author has describe : 

 In abstract section: Animal number in abstract section is similar in the method section. 

 In Introduction section, the author has revised the citation of the reference. The author has explained  a “gap” between glutamate excitotoxicity and kainic acid effects on OHSC in the background of this study.

 In the method section, the author has revised the exclusion criteria of this tissue study or sample studied.

 In the discussion section, the author has explained the strength and limitation of this study clearly. 

 In the conclusion section is clear and conclusive.

 Finally, this manuscript can be indexed.

Is the work clearly and accurately presented and does it cite the current literature?

Yes

If applicable, is the statistical analysis and its interpretation appropriate?

Yes

Are all the source data underlying the results available to ensure full reproducibility?

Yes

Is the study design appropriate and is the work technically sound?

Yes

Are the conclusions drawn adequately supported by the results?

Partly

Are sufficient details of methods and analysis provided to allow replication by others?

Yes

Reviewer Expertise:

Neurodegeneration and neurovascular

I confirm that I have read this submission and believe that I have an appropriate level of expertise to confirm that it is of an acceptable scientific standard.

# Reviewer report: Reviewer response for version 1

**DOI:** 10.5256/f1000research.139169.r166936

**Published:** 2023-04-14

**Authors:** Abdul Gofir

**Affiliations:** 1 Department of Neurology, Faculty of Medicine, Public Health and Nursing, Dr. Sardjito General Hospital, Universitas Gadjah Mada, Yogyakarta, Special Region of Yogyakarta, Indonesia

In the abstract, methods section, the paper said, “OHSC was obtained from two healthy wild types Wistar aged P10”. However, in the complete version, Methods, an overview of experiment section, first paragraph, the paper stated, “This study used three healthy wild type Wistar rats aged postnatal day 10…”. Which one is correct?

 Introduction, First Paragraph, 2 ^nd^ sentences

 The reference cited for this sentence (Mohd Sairazi) was a paper that explained about kainic acid excitotoxicity for the experimental model, while the sentence in your paper state that acute and chronic neurodegeneration is excitotoxicity without saying kainic acid in this paper. When we opened Mohd Sairazi's paper, their paper cited from Doble, 1999 the same sentence in their introduction paragraph. Please refer to the correct paper.

 Introduction (General Comment)

 The introduction section had explained clearly the importance of this study in examining kainic acid effects on the hippocampal area. It also stated clearly that the mechanism of neuronal death is glutamate excitotoxicity. However, there was still a “gap” between glutamate excitotoxicity and kainic acid effects on OHSC. Can you add more information to bridge the gap between these two pieces of information? (example: there is a necessity to make a correct animal model for glutamate excitotoxicity studies, etc)

 Methods, an overview of experiment section, First paragraph, Second sentence. Using Federer’s formula, could you state the number of treatments and the minimum sample size required for this study?

 Methods, Overview of the experiment, first paragraph, first sentence. This study used Wistar rats P10, while in the protocol cited for this study (Simoni & Yu, 2006) and (Opitz-Araya & Barria, 2010) used another rat model. Simoni & Yu used Sprague Dawley Rats P2-3, while Opitz-Araya & Barria used P5-P7. Could you add more information or references on choosing animals for your study?

 Methods, Overview of an experiment, second paragraph, the 7 ^th^ & 8 ^th^ sentence. Some slices were excluded because we're not very healthy macroscopically. This sentence was not clear enough to determine which slide was included or excluded; Hence, this exclusion could change the results of the study. Could you add more explanations on how you determined the tissue is not healthy and excluded from the study?

 Discussion Section, General Comments

 In this paper, you had explained clearly the comparison with other studies and explained what the mechanism behind your findings is. However, we didn’t find any explanation about strengths and weaknesses from your studies, because it will give further directions for other studies to fill any gaps that haven’t been answered in your study. Please add the strength and weaknesses, and what is the next direction for this topic

 Conclusions

 Usually in conclusions, we state what we found in our study without any citation. The second sentence showed a citation from Sattler & Tymianski, 2001, which had been explained clearly in the discussion section, the 8 ^th^ paragraph. Could you revise the conclusion section only include the conclusion from your study?

Is the work clearly and accurately presented and does it cite the current literature?

Yes

If applicable, is the statistical analysis and its interpretation appropriate?

Yes

Are all the source data underlying the results available to ensure full reproducibility?

Yes

Is the study design appropriate and is the work technically sound?

Yes

Are the conclusions drawn adequately supported by the results?

Partly

Are sufficient details of methods and analysis provided to allow replication by others?

Yes

Reviewer Expertise:

Neurodegeneration and neurovascular

I confirm that I have read this submission and believe that I have an appropriate level of expertise to confirm that it is of an acceptable scientific standard, however I have significant reservations, as outlined above.

# Response

**Published:** 2023-04-28

**Authors:** Machlusil Husna

**Affiliations:** Neurology Department, Faculty of Medicine, Universitas Brawijaya Malang, Indonesia

Mr Gofir,

 Thank you for your correction and suggestions. I would like to respond to your comments for my article

 Abstract (method section, line 1)

- the correct number of animal used is three. I will revise it in article.
- OHSC was obtained from three healthy wild type Wistar ratas aged P10. Healthy….

Introduction :

- Paragraph 1, line 4 : Sairazi citation.  I will also refer to the original paper.
  - Neurodegeneration mainly caused by acute and chronic excitotoxicity. The chronic excitotoxicity process can be found in Alzheimer’s disease (AD), Parkinson’s disease (PD), Huntington’s disease (HD), amyotropic lateral sclerosis (ALS), and temporal lobe epilepsy (TLE) ( Doble, 1999; Mohd. Sairazi *et al*, 2015)
- “gap” between glutamate excitotoxicity and kainic acid (paragraph 3, line 1)
  - “ Kainic acid is commonly used for glutamate excitotoxicity model. Damage due to kainic acid mainly occurs in the hippocampus. Such damage may be caused by the high density of kainic receptors in that area (Mihály, 2019; Zhang&Zhu, 2011).

Methods :

- Overview of experiments,  paragraph 1, line 2
  - “This study used three healthy wild type Wistar rats aged postnatal day 10 (P10), weight range 10-15 grams, regardless of sex. The sample size was decided from Federer’s formula (Ihwah et al., 2018). The number or treatments are two, and the result from the formula is 8, so we used 9 slices for each subgroup.”
- Overview of experiments, paragraph 2, line 7-8  
  - “….On day in vitro 13 (DIV 13), some slices were not very healthy macroscopically, so we exclude some slices for statistical analysis. The excluded slices conditions are : not getting thinner and transparent, also the color become whitish opaque or more darker”
- Overview of experiments,  paragraph 2, line 7 :  
  - I have mentioned the indicators for healthy slices macroscopically in discussion section (first paragraph, last sentence). However, I will add explanation in methods section as you suggested (paragraph 2, line 7).
    - “…The photos were processed with Adobe Photoshop ver.2020. We examined the healthiness of the slices macroscopically, using stereomicroscope.  The healty slices will look thinner and  more transparent, because the dead cells and tissue debris have disappeared. Another marker is we can see the different region of hippocampus clearly (Simoni & Yu, 2006). “
- Organotypic hippocampal slice culture procedure,  paragraph 2, line 1:
  -
    - “ Age recommendation to make OHSC for rat is P0-15 and P0-5 for mice (Muller, et al, 2001). We used P10 Wistar rat, because technically it is easier for us than younger rats. The brain size was not too small so it was easier to slice the hippocampus.”
- I will add explanation in discussion section (paragraph 1, line 10, new sentences) :
  - “ We could not use the unhealty slices, so we exclude it  for statistical analysis. We used only the healthy ones. The minimum requirement for comparation analysis is triplicate (we used 5 for calcium, 3 for MAP2, and 3 for GFAP). The statistical analysis still can be done, and the result was significant, in accordace with  the visual image obtained.”

Discussion :

- Strength and weakness : I will add  it in the discussion section (paragraph 8, new) so it will be clearer for readers
- “ The strength of this study is the demonstration of early damage of neuron and astrocyte by kainic acid, accompanied with high intracellular calcium expression. Only few prior studies have investigated it together. This early damage could lead to epileptogenesis. However,  we only measure the effect of excitotoxicity at one time only. Therefore, this study’s results can still be developed to further determine the course of neurons, astrocytes, as well as calcium in the epileptogenesis process and to find strategies to minimize excitotoxicitiy”

Conclusion : I will revise the conclusion as your suggestion

 The reduction of MAP2 and GFAP expression in this study indicates the loss of neurons and also astrocytes at the initial onset of exposure to kainic acid, which can be analogous to the deterioration of neuron and astrocyte function at the onset of insult leading to epilepsy.
